# Ion Concentration Polarization in Branched Microchannels: Effect of Membrane Thickness and Applied Voltage

**DOI:** 10.3390/membranes15090278

**Published:** 2025-09-17

**Authors:** Hirotada Hirama, Masanori Hayase

**Affiliations:** 1 Integrated Research Center for Self-Care Technology, National Institute of Advanced Industrial Science and Technology, Chiba 277-0882, Japan; 2 Faculty of Science and Technology, Tokyo University of Science, Chiba 278-8510, Japan

**Keywords:** microfluidics, particle concentration and separation, Nafion

## Abstract

Ion concentration polarization (ICP) is a promising electrokinetic technique for the concentration and separation of nanoparticles in microfluidic systems. In this study, we investigated how key parameters, including Nafion membrane thickness, applied voltage, and sample flow rate, influence the size of the ion depletion zone (IDZ), which is a critical factor governing ICP efficiency. Nafion membranes were fabricated via solution casting and patterning, producing non-uniform profiles with thinner centers and thicker edges. We found that thinner membranes (formed from 0.5 to 0.75 wt% solutions) led to IDZ widths 2–5 times greater than those of thicker membranes, likely due to nanogap formation at membrane-channel interfaces that enhanced ion transport. Additionally, higher applied voltages consistently enlarged the IDZ, consistent with the Nernst–Planck model, while increasing the flow rates reduced it. Notably, the combination of thin Nafion membranes and high voltage enabled stable IDZ formation, even at high flow rates. These findings offer important design insights for enhancing the performance and throughput of ICP-based nanoparticle manipulation devices.

## 1. Introduction

Ion concentration polarization (ICP) is an electrokinetic phenomenon caused by selective charge transport and is utilized in the concentration and separation of micro- and nanoparticles in microfluidic devices [1,2]. Hitherto, target samples that are concentrated by ICP include cells [3,4], proteins [5,6,7,8,9], DNA [10,11,12], and extracellular vesicles (EVs), such as exosomes [13]. In particular, EVs concentrated by ICP suffer less mechanical damage than that encountered when they are concentrated through conventional methods (e.g., ultracentrifugation) [13]. ICP is used as a separation technique for seawater desalination [14,15,16,17], separation of micellar neutral compounds from aqueous solutions [18], and isolation of micro- and nanoparticles from suspensions [19]. Rapid prototyping methods for ICP devices have been developed to facilitate their fabrication and testing at laboratory scale [20].

ICP facilitates the formation of an ion depletion zone (IDZ) that is electrically neutral and can therefore eliminate charged particles that are present in the microchannel (details of the principles are described below) [2]. The formed IDZ can be used to separate and concentrate micro- and nanoparticles. The size of the IDZ limits the concentration and separation throughput and is affected by the dimensions of the ion exchange membrane incorporated in the microchannel, as well as the applied voltage and sample flow rate. The effect of the applied voltage on ICP performance was previously investigated with respect to fluorescent dye manipulation using a microfluidic device comprising a Nafion membrane and branched microchannels [21]. In addition, the effect of the Nafion membrane thickness on ICP performance was investigated with respect to fluorescent dye manipulation using straight microchannels [22].

Previous studies have systematically investigated how key operating parameters affect ICP performance. Ko et al. reported that increasing the applied voltage enlarges the IDZ length, although excessively high voltages destabilize the IDZ boundary [7]. Kim et al. demonstrated that membrane thickness strongly influences ion-selective transport, with thicker membranes generally producing more stable ICP [22]. Phan et al. showed that increasing the applied voltage shifts the IDZ downstream, whereas higher flow rates reduce the IDZ size, requiring careful optimization of both parameters [21]. Jeon et al. compared thin (1.3 µm) and thick (5 µm) Nafion membranes and observed that thicker membranes produced larger particle repulsion distances under high voltages [19]. In addition, Kim et al. reported that thick polymer membranes enhance ICP by strengthening overlimiting conduction [4]. These results indicate that membrane thickness, applied voltage, and flow rate are critical factors governing IDZ/IDW formation.

In addition to these earlier works, several recent studies (2020–2025) have further advanced the quantitative understanding of ICP behavior under varied operational parameters. Kim et al. demonstrated rapid blood cell lysis enabled by voltage-driven ICP in Nafion microfluidic devices, highlighting the interplay between voltage and throughput [23]. Papadimitriou et al. introduced free-flow ICP focusing, showing that higher flow rates diminish the ion depletion zone and reduce enrichment efficiency [24]. Dang and Pham numerically investigated convergent channel geometries, reinforcing the effects of electric field and membrane placement on preconcentration [25]. Peramune et al. scaled ICP to millimeter-scale channels using Nafion microbead junctions, achieving high enrichment, albeit with Joule heating limitations at elevated flow rates [26]. Perera et al. realized rapid bacterial preconcentration in a paper-based ICP device, emphasizing passive capillary-driven operation for low-cost applications [27]. These studies collectively underscore the crucial roles of membrane thickness, applied voltage, and flow rate in ICP performance, and set the stage for our unique exploration of nanoparticle-focused ICP in branched microchannels with solution-cast Nafion membranes.

However, there has been no detailed investigation of the relationship between these parameters and the size of the IDZ thus far for the nanoparticle-targeted ICP phenomenon in branched microchannels. In addition, a Nafion membrane was fabricated by patterning and drying theNafion solution. The dimensional uniformity of the membrane produced via this method and its influence on ICP performance have not been investigated. Therefore, in this study, we investigated the effects of the aforementioned factors on IDZ size to determine the conditions suitable for efficient ICP operation.

The novelty of this study lies in demonstrating that thin Nafion membranes fabricated by solution casting can yield significantly larger IDWs than thicker membranes, in contrast with previous reports. This phenomenon is attributed to nanoscale gap formation at the membrane–channel interface, which enhances ion transport. Moreover, this work uniquely focuses on nanoparticle manipulation in branched microchannels, whereas most previous studies have investigated the ICP behavior in straight microchannels or with fluorescent dyes and biomolecules. These distinctions highlight the novelty of the present study and its contribution in advancing ICP-based nanoparticle manipulation in branched microchannels.

## 2. Theory

**Figure 1:**
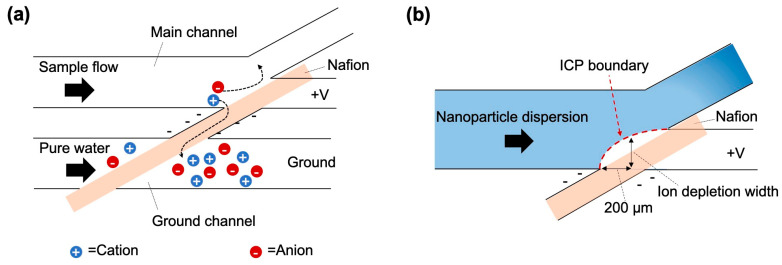
Schematic of microfluidic device used for ion concentration polarization (ICP). (**a**) ICP occurring in a microfluidic device owing to ion movement when voltage is applied. (**b**) Formed ICP boundary and ion depletion width.

ICP occurs within a microfluidic device consisting of two microchannels (the main channel for introducing the sample and the ground channel for introducing pure water) and a nanochannel connected via an ion-exchange membrane (Figure 1). When voltage is applied, the cations move from the main channel through the nanochannel to the ground channel (Figure 1a). Meanwhile, anions remained in the main channel, resulting in uneven ion composition within the main channel. To compensate for this ion imbalance, ions migrate from the surrounding solution, ultimately forming an electrically neutral IDZ, which in this study was recognized as the region where the fluorescence intensity was markedly reduced compared with the upstream baseline (Figure 1b). The IDZ formed in this manner can be used to remove or capture microparticles and nanoparticles. The size and shape of the IDZ vary significantly depending on the device design and operating conditions described below, which directly influences the concentration and separation performance.

## 3. Materials and Methods

### 3.1. Design and Fabrication of Microchannels for ICP

**Figure 2:**
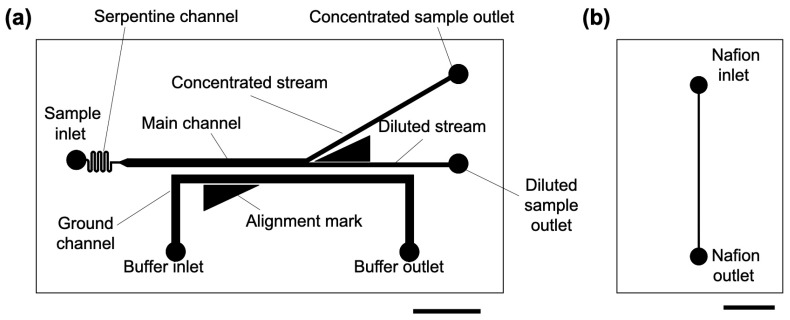
Microchannel design. (**a**) Microchannels for ion concentration polarization (ICP). (**b**) PDMS mold for patterning Nafion on a glass substrate. The scale bar represents 5 mm.

**Figure 3:**
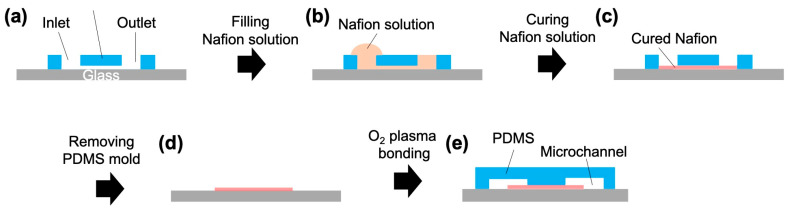
Fabrication procedure for the nanoparticle concentrator. (**a**) The PDMS mold was placed on a glass substrate. (**b**) The PDMS mold was filled with a Nafion solution. (**c**) Nafion solution was cured to fabricate a glass substrate with a Nafion membrane. (**d**) The PDMS mold was removed from the glass substrate. (**e**) The glass substrate with the Nafion membrane was bonded to a PDMS slab with a nanoparticle concentrator via O_2_ plasma treatment.

The ICP microchannel used in this study consisted of a main channel and ground channel with a width of 500 µm, branch channels (concentrated stream and diluted stream) with a width of 250 µm, and a serpentine channel (for flow stabilization) with a width of 100 µm (Figure 2a). Additionally, a straight-line microchannel with a width of 100 µm was designed as a mold for depositing the Nafion membrane (Figure 2b). The ICP microchannels were fabricated according to the method described by Mogi et al. [13]. The overview is as follows. First, conventional photolithography and soft lithography methods [28] were used to fabricate polydimethylsiloxane (PDMS)-based microchannels. Inlet and outlet holes were punched using a punch tool. For patterning the Nafion membrane, as shown in Figure 3, we attached a linear microchannel (PDMS mold) to a glass substrate (length: 7.6 cm, width: 5.2 cm, thickness: 0.8–1.0 mm, Matsunami Glass Ind., Ltd., Osaka, Japan) and filled the mold with a solution of Nafion (1100EW, Sigma-Aldrich, St. Louis, MI, Germany) diluted with ethanol (0.5, 0.75, 1, 2, 5 wt%) was filled into the mold. The filling was performed by dripping 70 µL of Nafion solution into the inlet and applying negative pressure from the outlet. The mold was placed on a hot plate at 100 °C for 10 min in the filled state, and a Nafion membrane was formed by drying and sintering. The PDMS mold was carefully peeled off to obtain a glass substrate with the formed membrane. Next, the glass substrate with the patterned Nafion membrane was bonded to an ICP microchannel via an oxygen plasma treatment, and the bond strength was enhanced under humid conditions (95 °C, 2 h, in an oven).

### 3.2. Microfluidic System

**Figure 4:**
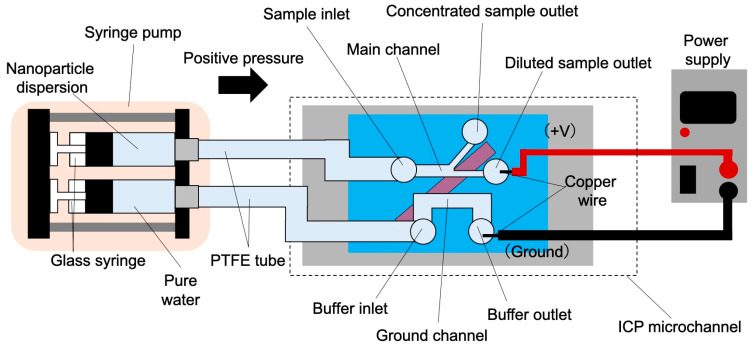
Conceptual diagram of microfluidic system.

For liquid delivery to the ICP microchannel, a polytetrafluoroethylene (PTFE) tube (inner diameter: 0.5 mm, outer diameter: 1.59 mm) was connected to a glass syringe (Figure 4). The nanoparticle dispersion solution was introduced from the sample inlet and ultrapure water was introduced from the buffer inlet. Liquid delivery was performed using a syringe pump (KDS-200, KD Scientific, Holliston, MA, USA). To simplify the experimental conditions, the flow rates in the main and ground channels were set equally (1, 5, and 15 μL/min). The nanoparticle dispersion solution used was prepared by suspending carboxyl-modified fluorescent polystyrene nanoparticles (average particle size 124.3 nm, density 1.03 g/cm^3^; Micromer^®^-redF, micromod Partikeltechnologie GmbH, Rostock, Germany), with the size measured previously in our earlier study [29], in water at a concentration of 9.5 × 10^10^ particles/mL, corresponding to approximately 98 µg/mL. The zeta potential of these nanoparticles was measured previously as −26.2 mV at pH 7 [29]. A DC-stabilized power supply (P4K-80M, Matsuda Precision Industry Co., Ltd., Shiga, Japan) was used to apply the voltage, which was connected to the diluted sample and buffer outlets. Although the applied voltage (20–100 V) could potentially induce electrolysis, no bubble formation was observed inside the microchannels during the experiments.

### 3.3. Measurement and Observation

To measure the Nafion membrane thickness, a laser microscope (LEXT-OLS 5000; Olympus Corporation, Tokyo, Japan) was used to scan the membrane in the short-axis direction. Field-emission scanning electron microscopy (FE-SEM, SM-7200F, JEOL Ltd., Tokyo, Japan) was used to observe the microstructure of the Nafion membrane. To observe the behavior of the nanoparticles within the ICP microchannel, a fluorescence microscope (BZ-X710, KEYENCE Corporation, Osaka, Japan) was used to acquire images. Fluorescence images were obtained under identical exposure settings across all experiments to ensure comparability, and the acquired color images were converted to grayscale before analysis to facilitate quantitative intensity profiling. Image analysis was performed using ImageJ software (version 1.54, National Institutes of Health, Bethesda, MD, USA).

The effects of various parameters, such as the applied voltage, Nafion membrane thickness, and flow rate, on the size of the IDZ were evaluated. The ion depletion width (IDW) was defined as an indicator of the IDZ size and determined from the fluorescence intensity distribution of the nanoparticle dispersion. For each experimental condition, the intensity profile was extracted along a line perpendicular to the main channel, located 200 μm upstream of the Nafion membrane (see Figure 1b). The IDW was defined based on the region of reduced fluorescence intensity in the profile.

## 4. Results and Discussion

### 4.1. Nafion Membrane Morphology

**Figure 5:**
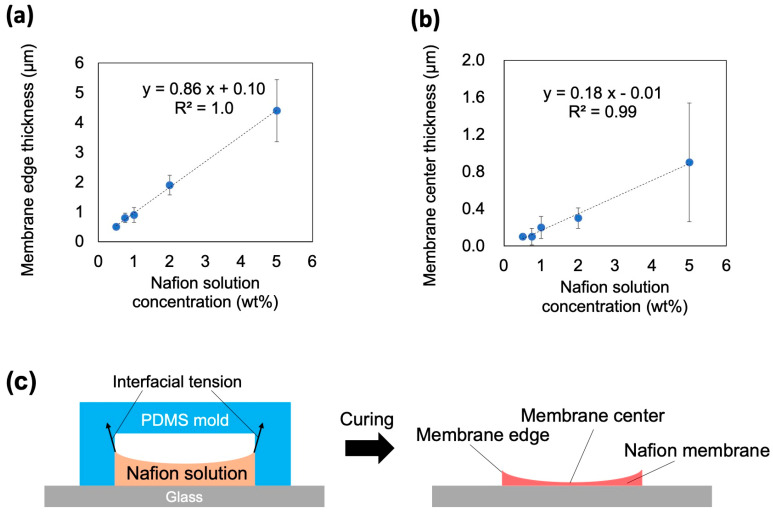
Thickness of Nafion membranes prepared from Nafion solutions of different concentrations. Relationship between Nafion solution concentration and thickness at membrane edge (**a**) and center (**b**) (n = 5). (**c**) Conceptual diagram of the cross-section in the short direction during the formation process of Nafion membranes.

One of the key factors significantly influencing the performance of ICP microchannels is the morphology of the Nafion membrane. In this section, we investigate the effect of the Nafion solution concentration on the membrane thickness and evaluate the structural characteristics of the membrane (Figure 5, Table 1). When the Nafion solution concentration is denoted as x wt%, the obtained membrane thickness is defined by the following equation:

(1) $$t\left(x\right)=\left(t_{c}\left(x\right),\;t_{e}\left(x\right)\right)$$

where t_c_(x) denotes the average membrane thickness at the membrane center and t_e_(x) denotes the average membrane thickness at the membrane edge. The membrane thickness exhibited a characteristic nonuniformity, with the central region being thinner and the edge region being thicker (Figure 5a,b). In this study, the “central region” was defined as the position 50 µm from the membrane edge toward the center. The membrane thickness showed an increasing trend as the concentration of Nafion solution increased. This phenomenon was attributed to the wetting and drying/hardening of the Nafion solution within the PDMS-based straight microchannel. Owing to the high wettability of the Nafion solution toward the PDMS surface, a meniscus-like membrane was formed during drying, resulting in thicker edges (Figure 5c). As shown in Figure 5, the membranes prepared from 0.5 wt% and 0.75 wt% Nafion solutions exhibited nearly identical thicknesses within the experimental variation. This similarity was also reflected in the IDW results (Figures 7 and 9), where both conditions showed comparable values.

### 4.2. Effects of Various Parameters on ICP

**Figure 6:**
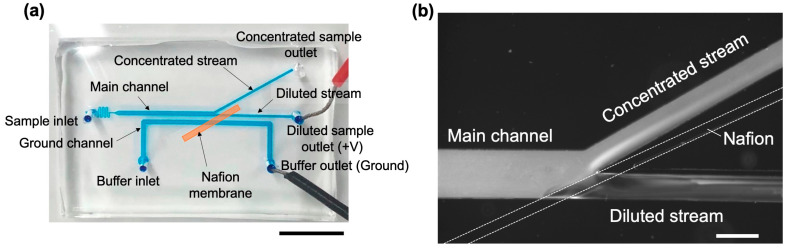
Fabricated microchannels. (**a**) Nafion membrane immobilized between PDMS-based microchannel and glass plate. The scale bar represents 5 mm. (**b**) Representative fluorescence image (converted to grayscale for clarity and quantitative analysis) showing ICP formation. The fluorescence intensity was reduced near the Nafion membrane, corresponding to the IDZ. The Nafion membrane was fabricated from 1 wt% Nafion solution. The flow rate was 1 µL/min, and the applied voltage was 100 V. The scale bar represents 500 μm.

When a voltage is applied to the fabricated ICP microchannel, ICP is generated within the channel, and the nanoparticles in the dispersion are mainly transported toward the concentrated stream side (Figure 6). As shown in Figure 6b, the ion depletion width (IDW) was determined from the fluorescence images of the nanoparticles. To further demonstrate the dependence on different experimental parameters, additional representative fluorescence images at varied voltages, membrane thicknesses, and flow rates are provided in the Supplementary Information (Figure S1). In this section, we evaluate the effects of the applied voltage, Nafion membrane thickness, and flow rate on ICP performance. As an indicator, we used IDW, which reflects the size of the formed IDZ.

#### 4.2.1. Effect of Voltage and Nafion Membrane Thickness

**Figure 7:**
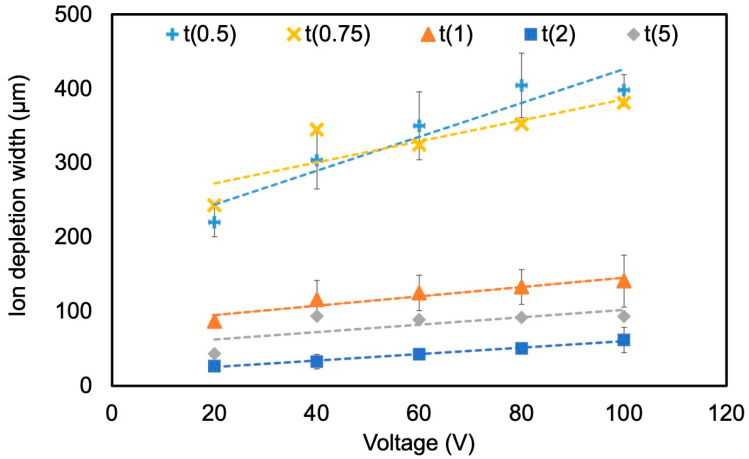
Effect of applied voltage on ion-depletion width. The flow rate was 1 μL/min. Error bars represent standard deviation. In some cases, the error bars were very small and overlapped the markers, making them visually indistinguishable. Each data point corresponds to five independent experiments.

**Figure 8:**
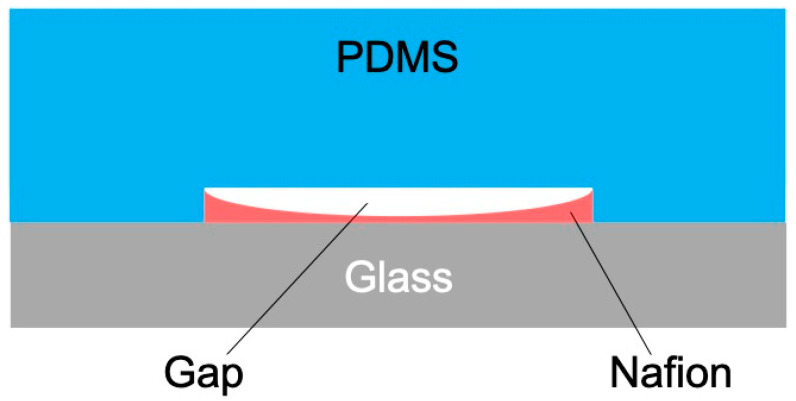
Cross-sectional diagram of Nafion embedded in the ICP microchannel.

The effect of the applied voltage on the size of the IDW in the ICP microchannel was investigated (Figure 7). Under conditions in which the thickness of the Nafion membrane was kept constant, it was confirmed that IDW tended to increase with increasing applied voltage. This relationship is attributed to the fact that cation transport through the ion-exchange membrane is promoted in proportion to the electric field strength. As shown in the following equation, the total ion transport flux N is expressed as the sum of the diffusion component and the electric field-driven component:

(2) $$N=-D\frac{dC}{dx}+µCE$$

where D is the diffusion coefficient, μ is the mobility, C is the ion concentration, and E is the applied electric field. This equation is based on the Nernst–Planck equation and indicates that ion transport driven by electric migration becomes dominant as the applied electric field increases [30]. In Kim et al.’s study, a phenomenon in which the conductivity of the desalting stream significantly decreased with increasing electric field strength was observed [14], which is consistent with the expansion of the IDZ. Furthermore, the influence of Nafion membrane thickness on the IDW was investigated. Under thin-membrane conditions (t(0.5), t(0.75)) prepared from 0.5 and 0.75 wt% Nafion solutions, IDW was found to be 2–5 times larger compared to thick-membrane conditions (t(1), t(2), t(5)). This difference is believed to be due to the morphology of the Nafion membrane formed during membrane deposition. In the membrane deposition method adopted in this study, a concave cross-sectional structure with a thinner central region and thicker edges was formed during the drying and curing processes (Figure 8). When such a membrane is bonded to a PDMS microchannel, nanometer-scale gaps are expected to be formed at the bonding interface. In relation to these structural characteristics, Kim et al. reported that patterned Nafion membranes achieve high selectivity and high-speed cation transport and that efficient ion movement due to nanoscale pore structures enhances ICP [22]. Based on this report and the behavior observed in this study, it is possible that the narrow gaps formed in the concave regions of the PDMS joint under thin-membrane Nafion conditions functioned as nanochannels, thereby enhancing ICP conductivity. However, the formation of the gaps was not explicitly mentioned in the report by Kim et al., and the interpretation in this study is a hypothetical consideration based on observations of the membrane shape and joint structure. However, under thick-membrane conditions (t(1) or greater), the gaps formed at the joint become wider, increasing the likelihood that they lose ion selectivity and function as conventional microchannels. Schoch et al. theoretically demonstrated that concentration polarization is suppressed in wide channels lacking ion selectivity [31], which is consistent with the observations in this study. In such cases, ion transport is likely confined to the Nafion membrane itself, and the expansion of IDW is expected to be limited. The above results suggest that the microstructure of the Nafion membrane, such as its thickness and shape, is a critical factor in the formation and maintenance of the ICP phenomenon. Further detailed analysis of the interface structure between the membrane and channel is expected to provide design guidelines for more efficient ICP devices.

Unlike previous reports, which generally indicated that thicker Nafion membranes enhance ICP stability and performance [7,19,22], our results demonstrated that thin membranes (0.5–0.75 wt%) prepared by solution casting produced IDWs 2–5 times larger than those of thicker membranes. This behavior can be explained by nanoscale gaps at the membrane–channel interface, formed during the casting and drying process, which provide additional ion transport pathways and lead to expansion of the IDZ.

#### 4.2.2. Effect of Flow Rate and Nafion Membrane Thickness

**Figure 9:**
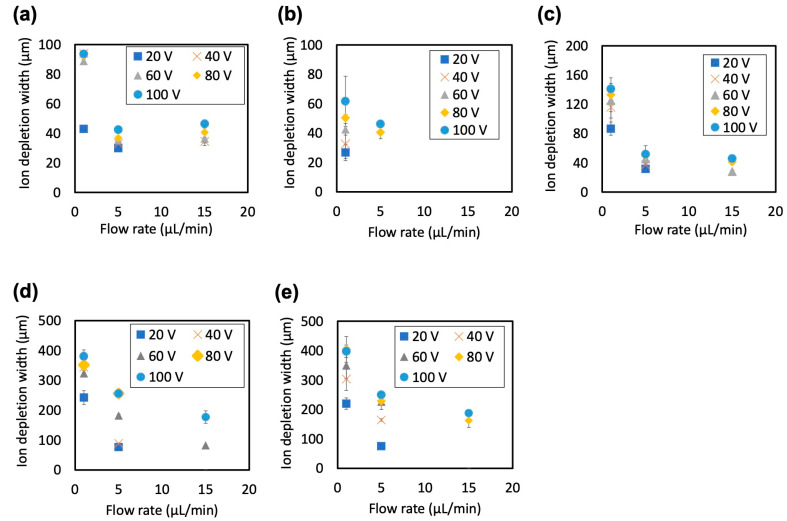
Effect of flow rate on ion depletion width. Nafion solution concentrations were (**a**) 5 wt%, (**b**) 2 wt%, (**c**) 1 wt%, (**d**) 0.75 wt%, and (**e**) 0.5 wt%. Error bars represent the standard deviation. In some cases, the error bars are very small and overlap with the markers, making them visually indistinguishable. Each data point corresponds to five independent experiments.

The effect of flow rate on the IDW formed in an ICP microchannel was investigated. Regardless of the thickness of the Nafion membrane, an increase in the flow rate consistently resulted in a decrease in IDW (Figure 9). In particular, at high flow rates (15 μL/min), the width of the formed IDZ narrowed, making stable observation difficult in certain cases. Additionally, the detection limit for IDW measurements is approximately 17 μm, making quantitative evaluation difficult below this threshold. Under conditions using relatively thick Nafion membranes (t(1), t(2), t(5)), the IDW was small, and it was difficult to form a reproducible IDZ. In contrast, in the ICP microchannel using relatively thin Nafion membranes (t(0.5), t(0.75)), it was possible to stably maintain a relatively wide IDZ even when the flow rate was increased by applying a high voltage. This is likely due to the narrow gap structure formed at the interface between the thin membrane and PDMS functioning as a nanochannel, thereby enhancing the ion transport efficiency, as mentioned earlier. These results indicate that the optimal combination of Nafion membrane thickness and applied voltage is critical for improving the throughput of the ICP devices. In particular, to form and maintain a sufficient IDZ even under high-flow-rateconditions, it is necessary to control the overall ion conductivity of the device by adjusting the membrane thickness. In the future, it is expected that optimization of the applied voltage and Nafion membrane thickness will lead to high-efficiency and high-throughput enhancement of nanoparticle concentration using ICP.

It should be noted that this study primarily focused on IDW as an indicator of ICP performance. Quantitative outlet concentration data were not obtained because the present device configuration was not optimized for such measurements. Clarifying the relationship between IDW and outlet concentrations would provide complementary insights, and we consider this an important direction for future studies.

#### 4.2.3. Sensitivity Analysis of Parameters

A simple sensitivity analysis was performed to further clarify the relative influence of the investigated parameters. For each parameter (applied voltage, membrane thickness, and flow rate), IDW changes were normalized to baseline values under fixed representative conditions (voltage, 20 V; flow rate, 1 μL/min; membrane thickness, t(2)). The results are summarized in Table 2. Detailed raw data for each parameter are provided in Supplementary Tables S1–S3 and the relative effects are shown in Supplementary Figure S2. According to the analysis, increasing the applied voltage from 20 to 100 V increased the IDW by approximately 2.3-fold (+130%). Reducing the membrane thickness from t(5) to t(0.5) resulted in a 4.4-fold increase in IDW (+1166%). In contrast, increasing the flow rate from 1 µL/min to 15 µL/min decreased the IDW by 0.1-fold (−173%). In this analysis, the fold change was defined as Fold = Y_max_/Y_baseline_, whereas % change was defined as %change = (Y_max_−Y_baseline_)/Y_baseline_ × 100%. Here, Y_max_ denotes the IDW at the maximum tested value of each parameter and Y_baseline_ denotes the IDW at the baseline condition. Because of this difference, fold change and % change are not always numerically proportional, although both metrics consistently indicate the same trend. These results quantitatively demonstrate the relative sensitivity of IDW to the tested parameters and suggest that thin membranes combined with high voltage are the most effective conditions for stable IDZ formation, even at higher flow rates.

## 5. Conclusions

In this study, we investigated how parameters such as Nafion membrane thickness, applied voltage, and flow rate affect IDW in an ICP microchannel. The results suggest that the Nafion membrane thickness is an important factor influencing IDW formation. In particular, under thin-membrane conditions of 0.5 and 0.75 wt%, IDW tended to be two to five times larger than that observed with relatively thicker membranes. This difference is thought to be due to the concave structure of the membrane formed during membrane deposition and the microscopic gaps formed at the interface between the PDMS channel and membrane, which function as nanochannels to assist ion transport. Furthermore, an increase in the applied voltage consistently contributed to the expansion of IDW, and this behavior was consistent with an ion transport model based on the Nernst–Planck equation. Additionally, while the IDW tended to decrease with increasing flow rate, under conditions using thin-membrane Nafion membranes, it was found that the effect could be partially suppressed by applying a high voltage. These results suggest that the combination of thin-membrane Nafion membranes and high voltage enables relatively stable IDZ formation, even under high flow rate conditions, making it an effective condition for improving ICP concentration performance. This could serve as a design guideline for future developments in high-efficiency processing and separation technologies for nanoparticles.

In addition, a simple sensitivity analysis was performed to quantitatively compare the relative effects of the tested parameters. The results confirmed that the applied voltage had the strongest positive effect on the IDW, whereas the flow rate reduced the IDW. Thin membranes prepared by solution casting provided the largest IDWs under high-voltage conditions, highlighting the importance of optimizing these parameters to achieve efficient and stable ICP performance.

## Figures and Tables

**Table 1:** Relationship between Nafion solution concentration and membrane thickness. x, t_c_(x), and t_e_(x) represent the Nafion solution concentration and thickness at the center and edge at concentration x wt%, respectively.

x	t_c_(x)	t_e_(x)
0.5	0.1 ± 0.02	0.5 ± 0.05
0.75	0.1 ± 0.09	0.8 ± 0.16
1	0.2 ± 0.12	0.9 ± 0.25
2	0.3 ± 0.11	1.9 ± 0.33
5	0.9 ± 0.64	4.4 ± 1.04

**Table 2:** Sensitivity summary of the effects of applied voltage, membrane thickness, and flow rate on IDW. Data were normalized to the baseline conditions (voltage, 20 V; flow rate, 1 μL/min; membrane thickness, t(2)). Fold change values represent the ratio of maximum to baseline IDW, while % change values were calculated relative to the baseline. Because of these different definitions, the fold change and % change are not always numerically proportional, but both indicate the same trend.

Parameter	Range Tested	Baseline	IDW Change (Fold)	IDW Change (%)	Slope (ΔY/ΔX)
Voltage	20 → 100 V	20 V	2.3×	+130	0.44 µm/V
Membrane thickness	t(5) → t(0.5)	t(2)	4.4×	+1166	−69 µm/wt%
Flow rate	1 → 15 µL/min	1 µL/min	0.1×	−173	−3.3 µm/(µL/min)

## Data Availability

The original contributions presented in this study are included in the article. Further inquiries can be directed to the corresponding author.

## Supplementary Materials

The following supporting information can be downloaded at: https://www.mdpi.com/article/10.3390/membranes15090278/s1, Figure S1: Representative fluorescence images of the nanoparticles under different experimental conditions; Figure S2: Relative effects of applied voltage, membrane thickness, and flow rate on IDW, expressed as % change compared with baseline values; Table S1: Effect of voltage on IDW; Table S2: Effect of membrane thickness on IDW; Table S3: Effect of flow rate on IDW.
