# Supplementary material for: Ion Concentration Polarization in Branched Microchannels: Effect of Membrane Thickness and Applied Voltage
Source: Membranes (Basel). 2025 Sep 17;15(9):278. doi: 10.3390/membranes15090278 (PMC12471405; doi:10.3390/membranes15090278)
Supplement: Supplementary file 1 [file membranes-15-00278-s001.zip › membranes-3834643-supplementary.pdf]

membranes

Supplementary Materials

Ion concentration polarization in branched microchannels: Effect of membrane thickness and applied voltage

Hirotada Hirama [1,*], Masanori Hayase [2]

[1] Integrated Research Center for Self-Care Technology, National Institute of Advanced Industrial Science and Technology, Chiba 277-0882, Japan
[2] Faculty of Science and Technology, Tokyo University of Science, Chiba 278-8510, Japan
* Correspondence: h.hirama@aist.go.jp

 https://doi.org/10.3390/membranes15090278

Table S1: Effect of voltage on IDW.

Voltage (V)	IDW (μm)	Fold vs 20 V	∆ IDW (%)
20	26.8	1.0	0
40	32.5	1.2	21
60	42.5	1.6	59
80	50.4	1.9	88
100	61.6	2.3	130

Table S2: Effect of membrane thickness on IDW.

Membrane thickness	IDW (μm)	Fold vs t(2)	∆ IDW (%)
t(0.5)	405	4.4	1166
t(0.75)	352	3.8	970
t(1)	133	1.4	153
t(2)	50.4	0.5	-156
t(5)	92.1	1.0	0

Table S3: Effect of flow rate on IDW.

Flow rate (μL/min)	IDW (μm)	Fold vs 1 μL/min	∆ IDW (%)
1	50.4	1.00	0
5	40.5	0.80	-37
15	4	0.1	-173

(a)

(b)

(c)

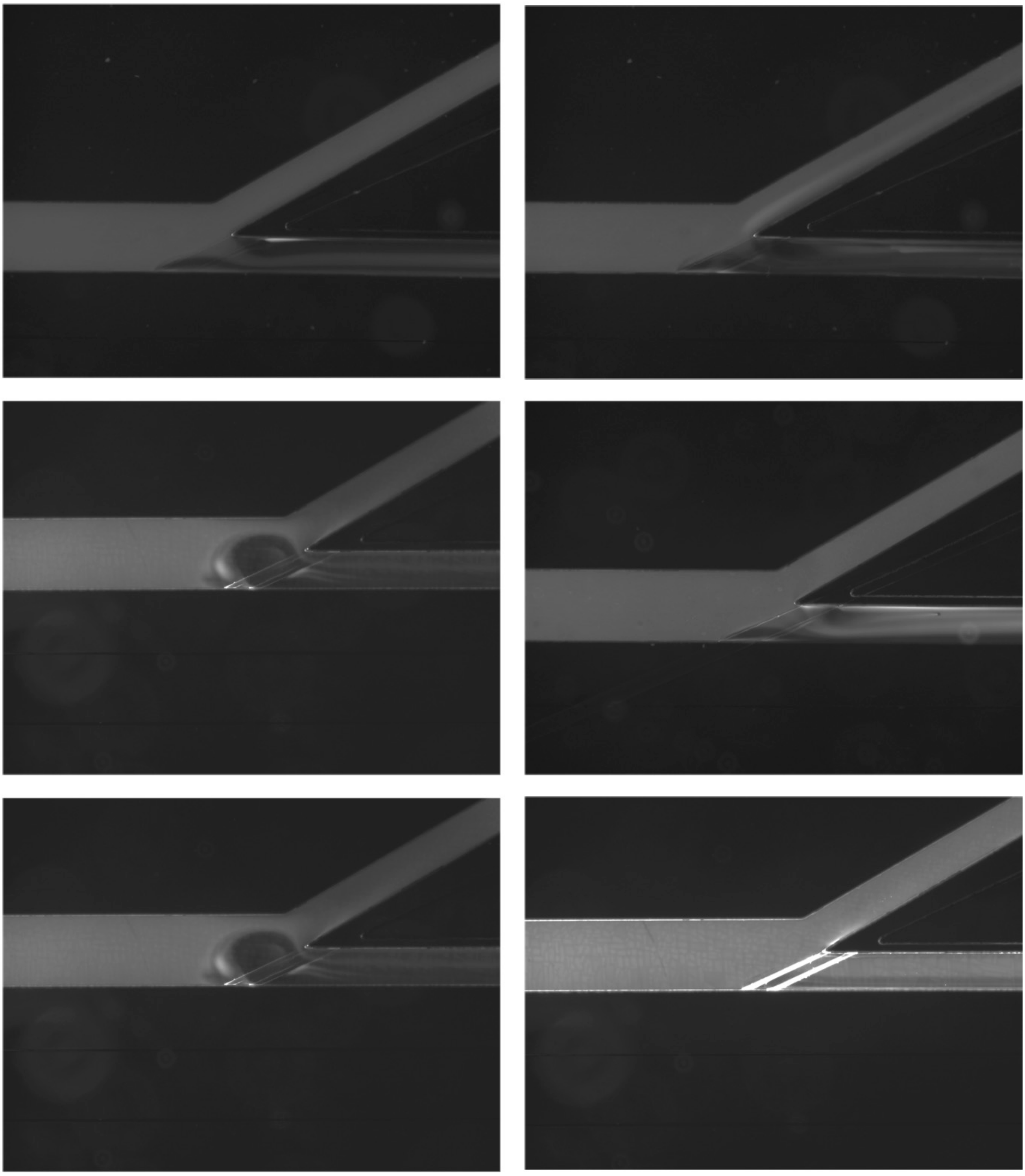

Figure S1: Representative fluorescence images of the nanoparticles under different experimental conditions. (a) Applied voltage: 20 V (left) and 100 V (right) with membrane thickness t(1) and flow rate of 1 μL/min. (b) Membrane thickness: t(0.5) (left) and t(5) (right) at 100 V and 1 μL/min. (c) Flow rate: 1 μL/min (left) and 15 μL/min (right) with membrane thickness t(0.5) at 100 V.

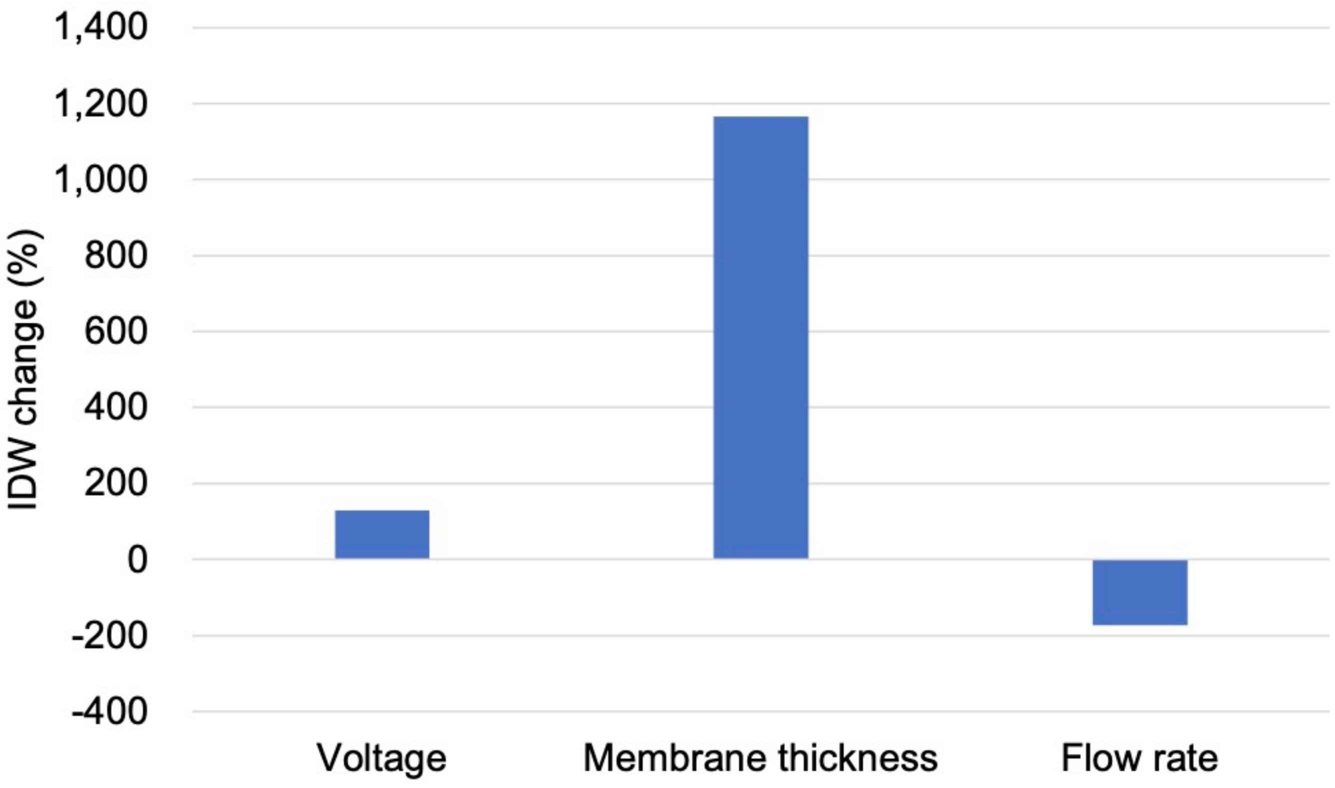

Figure S2: Relative effects of applied voltage, membrane thickness, and flow rate on IDW, expressed as percentage change compared with baseline values.
